# Perceived anthropomorphism of AI and belongingness need: the sequential roles of liking and perceived emotional support

**DOI:** 10.3389/fpsyg.2026.1842149

**Published:** 2026-07-23

**Authors:** Jinrui Tian, Boxuan Li, Ronghua Zhang, Xiaoliang Liu

**Affiliations:** 1 School of Marxism, Wuhan University, Wuhan, China; 2 Laboratory for Youth Values Education, Wuhan University, Wuhan, China

**Keywords:** belongingness need, human–AI interaction, liking of AI, perceived anthropomorphism of AI, perceived emotional support, social surrogate

## Abstract

**Introduction:**

This study examines how perceived anthropomorphism of artificial intelligence (AI) is associated with users’ belongingness need through liking of AI and perceived emotional support. Drawing on the three-factor theory of anthropomorphism, the Computers as Social Actors paradigm, social support theory, and the social surrogate hypothesis, we propose that users’ human-like perceptions of AI are linked to belongingness-related motivation through a sequential socioemotional process.

**Methods:**

Survey data were collected from 473 college students with prior experience using AI tools or conversational AI assistants. Participants completed measures of perceived anthropomorphism of AI, liking of AI, perceived emotional support, and belongingness need.

**Results:**

Confirmatory factor analysis showed that the hypothesized four-factor model fit the data better than alternative models, providing preliminary evidence for the empirical distinctiveness of the focal constructs. Liking of AI and perceived emotional support each served as significant mediators in this association. In addition, the sequential indirect path from perceived anthropomorphism to liking of AI, perceived emotional support, and belongingness need was significant.

**Discussion:**

These findings suggest that anthropomorphic perceptions of AI are related to users’ belongingness-related motivation through layered affective and socioemotional processes. The study contributes to human–AI interaction research by distinguishing liking of AI from perceived emotional support and by clarifying how anthropomorphic AI perceptions are associated with users’ social–emotional experiences. The findings should be interpreted within the limits of the cross-sectional design and the outcome measure: belongingness need reflects users’ desire for acceptance and connection rather than actual belongingness satisfaction.

## 1 Introduction

Artificial intelligence (AI) refers to intelligence exhibited by machines, distinct from natural human intelligence (Poole et al., 1998). With advances in computing power and data availability, conversational agents have moved beyond laboratory settings and become integrated into smartphones and computers as chatbots and voice assistants, providing on-demand information, scheduling, and entertainment services (Følstad and Brandtzæg, 2017; Hoy, 2018). These dialogic systems, when designed with greater human-likeness and utility, are more likely to receive favorable user evaluations and promote continued engagement (Fernandes and Oliveira, 2021). Understanding why users like, trust, and increasingly rely on such AI has emerged as a critical focus in human–computer interaction (HCI) research.

According to the need to belong theory, humans possess an innate motivation to form and maintain positive and lasting social bonds (Baumeister and Leary, 1995). When this need is thwarted, individuals may experience loneliness and turn to “social surrogates,” such as favorite television shows or digital pets, as alternative sources of socioemotional experience (Derrick et al., 2009). In the rapidly expanding context of conversational AI, this substitution effect may become more salient: always-available social and companion chatbots can serve as sources of companionship and emotional support (Ta et al., 2020; Brandtzaeg et al., 2022).

Perceived anthropomorphism plays a pivotal psychological role in this surrogate process. Individuals tend to attribute human-like qualities to non-human agents (Waytz et al., 2010). According to the three-factor model proposed by Epley et al. (2007), anthropomorphic tendencies are driven by knowledge of human characteristics, effectance motivation, and sociality motivation. When social motivations are activated due to a lack of connection, users are more likely to perceive computers, robots, or chat agents as “quasi-human companions.” The resulting liking of AI and perceived emotional support may serve as psychological channels through which anthropomorphic perceptions of AI are associated with belongingness-related motivation. Enhancing anthropomorphic cues such as appearance or emotional expression, can significantly boost users’ trust in, empathy toward, and positive attitudes toward AI agents (Kim and Sundar, 2012).

Over the past decade, general-purpose conversational AIs have largely been evaluated in HCI through an instrumental lens, with algorithmic accuracy, response speed, task success rate, and productivity gains treated as central indicators of system value (Luger and Sellen, 2016; Hoy, 2018; OpenAI, 2023). Although this “performance paradigm” has contributed to important advances in natural language processing and dialogue systems, it has paid less attention to users’ emotional experiences and social motivations, including how interactions with companion chatbots may be experienced as sources of companionship and emotional support (Ta et al., 2020). More recently, researchers have adopted a more human-centered perspective, showing that some users develop personalized relationships with social chatbots that they understand in ways resembling human friendship (Brandtzaeg et al., 2022). Even so, this area remains at an early stage, and the psychological processes through which such relationships emerge and influence users are still not fully understood. Relatedly, Singh (2022) found that users’ emotional attachment to voice-based AI interfaces, such as smart assistants, significantly increased satisfaction and repurchase intentions, suggesting that emotionally driven human–AI relationships may have lasting behavioral consequences.

The theoretical logic of the present study is organized across three levels. First, the need to belong theory provides the motivational background, suggesting that individuals have a fundamental desire for acceptance, connection, and stable social bonds. Second, the three-factor theory of anthropomorphism explains why users may attribute human-like characteristics to AI, especially when social motivation is salient. Third, the CASA paradigm and media equation theory explain why perceived human-like cues in AI can elicit social and affective responses similar to those found in interpersonal interaction. Building on this foundation, liking of AI is conceptualized as an immediate affective appraisal of the AI as a social interaction partner, whereas perceived emotional support reflects a more relational interpretation of AI responses as caring, understanding, or comforting. Thus, the social surrogate hypothesis is not treated as a separate competing theory, but as a broader explanatory lens suggesting that non-human agents may sometimes serve as alternative sources of socioemotional experience. This layered framework allows us to specify a sequential pathway from perceived anthropomorphism to liking, perceived emotional support, and belongingness need.

The present study aims to examine whether perceived anthropomorphism of AI is associated with users’ belongingness need through two socioemotional mechanisms: liking of AI and perceived emotional support. Rather than assuming that AI directly fulfills belongingness needs, we conceptualize belongingness need as a motivational tendency reflecting users’ desire for acceptance, connection, and social inclusion. Drawing on anthropomorphism theory and the CASA paradigm, we argue that when AI is perceived as more human-like, users may respond to it with stronger liking. This initial affective appraisal may further shape the extent to which users interpret AI responses as emotionally supportive. In turn, these socioemotional responses may be associated with stronger belongingness-related psychological experiences. By testing this sequential mediation model, the study seeks to clarify how anthropomorphic perceptions of AI are linked to users’ social–emotional needs in contemporary human–AI interaction.

### 1.1 Perceived anthropomorphism of AI and belongingness need

The need to belong refers to individuals’ fundamental motivation to seek acceptance, connection, and stable social bonds (Baumeister and Leary, 1995). Importantly, belongingness need reflects the strength of this social motivation rather than the extent to which the need has already been satisfied. When individuals experience a stronger desire for social connection, they may become more attentive to social cues in their surrounding environment, including cues embedded in technological agents.

Anthropomorphism refers to the attribution of human-like characteristics, intentions, or mental states to non-human agents (Epley et al., 2007; Waytz et al., 2010). According to the three-factor theory of anthropomorphism, sociality motivation is one important factor that increases people’s tendency to perceive non-human agents as human-like. In the context of AI interaction, users who perceive AI as more human-like may be more likely to treat it as a socially meaningful interaction partner rather than merely as a functional tool. The CASA paradigm and media equation theory further suggest that people often respond socially to computers and media technologies when these systems present social cues, even when users consciously know that the systems are non-human (Reeves and Nass, 1996; Nass and Moon, 2000).

On this basis, perceived anthropomorphism of AI may be positively associated with users’ belongingness need. This association does not necessarily imply that AI satisfies belongingness needs. Rather, it suggests that users who perceive AI as more human-like may also report stronger belongingness-oriented psychological tendencies, such as a stronger desire for acceptance, connection, and interpersonal inclusion. Therefore, we propose the following hypothesis:

*H1*: Perceived anthropomorphism of AI is positively associated with belongingness need.

### 1.2 The mediating role of liking of AI

Perceived anthropomorphism may be linked to belongingness need partly through users’ liking of AI. Liking refers to a positive affective appraisal of the AI as friendly, pleasant, and approachable. In human–AI interaction, anthropomorphic cues can make an AI system appear warmer, more socially present, and more similar to an interpersonal partner, thereby increasing users’ positive affective evaluations of the system (Aggarwal and McGill, 2007; Bartneck et al., 2009; Waytz et al., 2014).

The CASA paradigm provides a theoretical explanation for this process. When AI displays human-like cues, users may apply social scripts and interpersonal expectations to the interaction, leading them to evaluate the AI in a manner similar to how they evaluate human interaction partners. Thus, perceived anthropomorphism may increase liking because human-like AI is more easily interpreted as socially approachable and emotionally engaging.

Liking of AI may, in turn, be associated with belongingness need. In interpersonal contexts, liking is often an early affective basis for social approach, interaction willingness, and perceived closeness. Similarly, in AI interaction, users who like an AI system may be more willing to interact with it, attend to its responses, and experience the interaction as socially meaningful. Therefore, liking may serve as an affective bridge linking perceived anthropomorphism to belongingness-related motivation. Accordingly, we propose:

*H2*: Liking of AI mediates the association between perceived anthropomorphism of AI and belongingness need.

### 1.3 The mediating role of perceived emotional support and the sequential mediation pathway

In addition to liking, perceived emotional support may also explain the association between perceived anthropomorphism and belongingness need. Emotional support refers to the perception that an interaction partner provides understanding, encouragement, comfort, and care (Cutrona and Russell, 1990; Cohen, 2004). In human–AI interaction, users may perceive AI responses as emotionally supportive when the AI appears to understand their concerns, respond empathically, or provide comforting feedback.

Anthropomorphic perceptions may strengthen such support-related interpretations. When AI is perceived as more human-like, users may be more likely to attribute warmth, concern, and responsiveness to its replies. This does not mean that AI objectively possesses human emotions or genuine care. Rather, it indicates that users may subjectively interpret AI responses as supportive when the system is experienced as socially present and human-like. Therefore, perceived emotional support may serve as a socioemotional mechanism linking anthropomorphic perception to belongingness need. We propose:

*H3*: Perceived emotional support mediates the association between perceived anthropomorphism of AI and belongingness need.

Furthermore, we propose a sequential pathway in which liking of AI precedes perceived emotional support. Theoretically, liking represents a relatively immediate affective appraisal of the AI as pleasant and approachable, whereas perceived emotional support involves a more relational interpretation of the AI’s responses as caring, understanding, or comforting. In other words, users may first develop a positive affective orientation toward an anthropomorphized AI; this liking may then increase their willingness to engage with the AI, attend to its responses, and interpret those responses as emotionally supportive.

This order is also consistent with social support theory and the social surrogate hypothesis. People are more likely to perceive support from agents they evaluate positively and experience as socially acceptable. In the AI context, liking may make users more receptive to AI-generated responses, thereby increasing the likelihood that these responses are perceived as emotionally supportive. Such perceived emotional support may then be associated with stronger belongingness-related psychological tendencies. Therefore, we propose:

*H4*: Liking of AI and perceived emotional support sequentially mediate the association between perceived anthropomorphism of AI and belongingness need, such that perceived anthropomorphism is associated with greater liking of AI, which is subsequently associated with greater perceived emotional support and, in turn, higher belongingness need.

### 1.4 The present study

Overall, research across various domains has consistently shown that people tend to respond to computers and AI systems with human-like characteristics in socially oriented ways (Nass and Moon, 2000). Early studies also attempted to facilitate long-term relationships between users and virtual agents with personalized traits (Bickmore and Picard, 2005). A large body of empirical research has shown that anthropomorphic design is associated with users’ trust, satisfaction, and willingness to use these systems (Blut et al., 2021). At the same time, an increasing number of studies have begun to examine whether anthropomorphic technologies are psychologically relevant to users’ socioemotional experiences. For example, under certain conditions, engaging with anthropomorphized technologies may be associated with reduced psychological discomfort in socially threatening contexts (Mourey et al., 2017). Related literature also indicates that conversational AI has potential as a perceived source of social support (Følstad and Brandtzæg, 2017).

However, current findings are not entirely consistent. Some studies have failed to find evidence that human–AI interactions significantly reduce users’ desire for human contact, arguing that technology cannot fully replace real interpersonal connections in meeting social needs (Christoforakos and Diefenbach, 2023). This suggests that the socioemotional relevance of AI has important limitations, and that the quality of human–AI relationships still differs from human–human relationships.

Existing literature also reveals several gaps. First, many early studies mainly focused on anthropomorphism in terms of appearance and expressive behaviors, emphasizing user acceptance, trust, and interaction quality, while paying relatively less attention to belongingness-related psychological processes. Second, much of the research has concentrated on AI in specific contexts, leaving the socioemotional mechanisms of frequently used AI tools and conversational AI assistants underexplored. Finally, the psychological processes through which anthropomorphic perceptions are linked to users’ belongingness-related motivation remain insufficiently understood. In particular, the sequential pathway involving liking of AI and perceived emotional support requires further empirical examination.

It is also important to note that anthropomorphic design poses challenges in terms of degree. Overly realistic or excessive human-like cues may trigger discomfort or the so-called “uncanny valley” effect, and may also lead users to overestimate AI’s relational capacities (Mori et al., 2012). Therefore, research should carefully examine both the socioemotional value and the boundaries of AI anthropomorphism.

## 2 Materials and methods

### 2.1 Participants and procedure

A total of 559 undergraduate students were recruited to participate in this study. Participants were aged between 18 and 24 years (M = 19.01, SD = 2.00). Among them, 49.55% were male and 50.45% were female. Participants came from diverse academic backgrounds, including science and engineering, medicine, arts and humanities, and social sciences.

Data were collected in May 2025 during in-person classroom sessions using paper-based questionnaires. The study targeted students who had prior experience using AI tools or conversational AI assistants. Before completing the questionnaire, participants were instructed to answer all AI-related items with reference to the AI tool or conversational AI assistant that they used most frequently. This instruction was intended to ensure that participants evaluated a concrete AI interaction object rather than responding to the items in a purely abstract manner.

After data screening, 86 responses were excluded because participants failed attention-check items, completed the questionnaire in an unreasonably short time, or did not meet the AI-use eligibility criterion. The final sample consisted of 473 valid participants, including 234 male participants (49.47%) and 239 female participants (50.53%). Among the valid participants, 312 participants (65.96%) reported using AI tools or conversational AI assistants on a daily basis.

All participants provided written informed consent prior to participation. They were informed that participation was voluntary, that their responses would remain anonymous, and that they could withdraw from the study at any time without penalty.

### 2.2 Measures

For all AI-related measures, participants were asked to evaluate the AI tool or conversational AI assistant that they used most frequently. Accordingly, the term “AI” in the following measures refers to this self-selected, frequently used AI interaction object.

#### 2.2.1 Perceived anthropomorphism scale

Perceived anthropomorphism of AI was measured using the scale developed by Waytz et al. (2010). The scale consists of five items assessing the extent to which participants attributed human-like qualities, intentions, or mental capacities to the AI tool or conversational AI assistant they evaluated. A sample item is “I believe this AI thinks and behaves like a human.” Items were rated on a 7-point Likert scale ranging from 1 (strongly disagree) to 7 (strongly agree). Higher scores indicated stronger perceived anthropomorphism of the evaluated AI. In the present study, the scale demonstrated high internal consistency, Cronbach’s *α* = 0.90.

#### 2.2.2 Liking of AI scale

Liking of AI was assessed using the likeability subscale of the Godspeed Questionnaire developed by Bartneck et al. (2009). This subscale includes five semantic differential items, such as “unlikable–likable” and “unfriendly–friendly.” Participants rated their perception of the AI tool or conversational AI assistant they used most frequently on a 5-point scale. Higher scores indicated a more positive affective evaluation of the evaluated AI. In the present study, the scale showed excellent internal consistency, Cronbach’s α = 0.93.

#### 2.2.3 Perceived emotional support scale

Perceived emotional support from AI was measured using items adapted from Methot et al. (2016), with reference to emotional support theory (Schaefer et al., 1981; Mossholder et al., 2005). The items were adapted to the AI context by replacing references to interpersonal sources of support with the AI tool or conversational AI assistant evaluated by participants. Sample items include “When I encounter problems, this AI assistant shares personalized examples to offer a different perspective” and “This AI assistant provides me with encouragement and emotional support.” Participants responded on a 5-point Likert scale ranging from 1 (strongly disagree) to 5 (strongly agree). Higher scores indicated stronger perceived emotional support from the evaluated AI. This scale captures users’ subjective perception of support rather than implying that AI possesses genuine emotions or human-like care. In the present study, the scale demonstrated high internal consistency, Cronbach’s *α* = 0.93.

#### 2.2.4 Need for belonging scale

Belongingness need was measured using the Chinese version of the Need to Belong Scale translated and revised by Ma (2008), based on the original conceptualization of the need to belong as a fundamental motivation for acceptance and social connection. The scale includes items such as “I want others to accept me” and “I do not like being alone.” Items 1, 2, and 6 were reverse-coded. Participants responded on a 7-point Likert scale ranging from 1 (not at all true of me) to 7 (very true of me). Higher scores indicated a stronger need for acceptance, connection, and belonging. Importantly, this measure assesses the strength of belongingness need rather than the extent to which the need has been satisfied. In the present study, the internal consistency of the scale was acceptable, Cronbach’s α = 0.73.

### 2.3 Analysis method

All statistical analyses were conducted using SPSS 27.0. First, descriptive statistics and Pearson correlation analyses were performed to examine the associations among perceived anthropomorphism of AI, liking of AI, perceived emotional support, and belongingness need. Second, independent-samples *t*-tests were used to examine whether the main study variables differed by gender. Third, to test the hypothesized indirect association between perceived anthropomorphism of AI and belongingness need through liking of AI and perceived emotional support, we used the PROCESS macro for SPSS. Specifically, Model 6 was applied to estimate the sequential mediation model. Bootstrap resampling with 5,000 samples was used to generate 95% confidence intervals for the indirect effects. Indirect effects were considered statistically significant when the 95% bootstrap confidence interval did not include zero. Because the study used cross-sectional self-report data, the mediation analyses were interpreted as tests of theoretically specified indirect associations rather than evidence of causal or temporal ordering.

## 3 Results

### 3.1 Measurement validity and common method bias

Before testing the hypothesized mediation model, confirmatory factor analysis (CFA) was conducted to examine the measurement validity and discriminant validity of the four focal constructs: perceived anthropomorphism of AI, liking of AI, perceived emotional support, and belongingness need. The hypothesized four-factor measurement model, in which all items loaded onto their corresponding latent constructs, showed better relative fit than the alternative models, χ^2^ = 1532.87, df = 269, χ^2^/df = 5.70, CFI = 0.838, TLI = 0.820, RMSEA = 0.100, SRMR = 0.111.

To further evaluate whether the focal constructs were empirically distinguishable, the hypothesized four-factor model was compared with alternative models. Given the relatively high correlation between liking of AI and perceived emotional support, we first tested a three-factor model in which these two constructs were combined into one factor. This model showed poorer fit than the four-factor model, χ^2^ = 2164.28, df = 272, χ^2^/df = 7.96, CFI = 0.758, TLI = 0.733, RMSEA = 0.121, SRMR = 0.116. We also tested a single-factor model in which all measurement items were loaded onto one common factor. The single-factor model showed poor fit, χ^2^ = 3675.78, df = 275, χ^2^/df = 13.37, CFI = 0.565, TLI = 0.525, RMSEA = 0.162, SRMR = 0.138. These model comparisons suggested that the four-factor structure fit the data better than the alternative models and provided preliminary support for the empirical distinctiveness of the focal constructs, including the distinction between liking of AI and perceived emotional support. The CFA results and model comparisons are presented in Table 1.

**Table 1:** Results of confirmatory factor analyses.

Model	χ^2^	df	χ^2^/df	CFI	TLI	RMSEA	SRMR
Four-factor measurement model	1532.87	269	5.70	0.838	0.820	0.100	0.111
Three-factor model: liking of AI and perceived emotional support combined	2164.28	272	7.96	0.758	0.733	0.121	0.116
Single-factor model	3675.78	275	13.37	0.565	0.525	0.162	0.138

The four-factor model included perceived anthropomorphism of AI, liking of AI, perceived emotional support, and belongingness need as separate latent constructs. CFI, comparative fit index; TLI, Tucker–Lewis index; RMSEA, root mean square error of approximation; SRMR, standardized root mean square residual.

Common method bias was also examined because all variables were measured using self-report questionnaires collected from the same participants, a design feature that may increase the risk of common method variance (Podsakoff et al., 2003). Harman’s single-factor test showed that six factors had eigenvalues greater than 1, and the first factor accounted for 32.7% of the total variance, which was below the commonly used threshold of 40%. In addition, the poor fit of the single-factor CFA model provided further evidence that the observed relationships were unlikely to be fully attributable to a single common method factor. Taken together, these results suggest that common method bias was not severe enough to invalidate the subsequent analyses. Nevertheless, because the present study relied on cross-sectional self-report data, potential common method bias remains a methodological limitation and is further discussed in the Limitations section.

### 3.2 Means, standard deviations, and correlation matrix of variables

Descriptive statistics and Pearson correlation analyses were conducted to examine the associations among the main study variables, and the results are presented in Table 2. Descriptive statistics showed that participants reported moderate levels of perceived anthropomorphism of AI (M = 3.73, SD = 1.17), liking of AI (M = 3.25, SD = 0.92), perceived emotional support (M = 3.20, SD = 0.91), and belongingness need (M = 4.54, SD = 0.66).

**Table 2:** Means, standard deviations, and correlations among variables.

Variable	*M*	*SD*	1	2	3	4
1. Perceived anthropomorphism	3.73	1.17	—			
2. Liking of AI	3.25	0.92	0.57^***^	—		
3. Perceived emotional support	3.20	0.91	0.53^***^	0.71^***^	—	
4. Belongingness need	4.54	0.66	0.34^***^	0.39^***^	0.42^***^	—

*N* = 473. *p < 0.05, **p < 0.01, ***p < 0.001.

Correlation results revealed a significant positive correlation between perceived anthropomorphism of AI and belongingness need (*r* = 0.34, *p* < 0.001), suggesting that individuals who tended to attribute stronger human-like qualities to the AI tool or conversational AI assistant they used most frequently also reported stronger belongingness need. This result provided preliminary correlational support for Hypothesis 1. Additionally, perceived anthropomorphism of AI was significantly and positively correlated with liking of AI (*r* = 0.57, *p* < 0.001) and perceived emotional support (*r* = 0.53, *p* < 0.001), indicating that stronger perceptions of AI anthropomorphism were associated with greater liking of AI and stronger perceived emotional support from AI.

Liking of AI also showed a significant positive correlation with belongingness need (*r* = 0.39, *p* < 0.001), suggesting that users who reported greater liking of AI also tended to report stronger belongingness need. Furthermore, liking of AI was highly positively correlated with perceived emotional support (*r* = 0.71, *p* < 0.001), indicating that users who liked AI more were also more likely to perceive stronger emotional support from it. Although the correlation between liking of AI and perceived emotional support was relatively high, the CFA results reported in Section 3.1 supported their empirical distinctiveness. A significant positive correlation was also found between perceived emotional support and belongingness need (*r* = 0.42, *p* < 0.001), suggesting that users who perceived stronger emotional support from AI also tended to report stronger belongingness need.

Moreover, independent-samples t-tests revealed no significant gender differences across the key psychological variables, including perceived anthropomorphism of AI, liking of AI, perceived emotional support, and belongingness need, ps > 0.05. These results indicated that gender was not significantly associated with the main psychological constructs under investigation. In summary, the correlation results provided preliminary support for the hypothesized associations among perceived anthropomorphism of AI, liking of AI, perceived emotional support, and belongingness need, with the positive correlation between perceived anthropomorphism and belongingness need specifically supporting Hypothesis 1 at the bivariate level.

### 3.3 Sequential mediation analysis

The results of the sequential mediation model are shown in Table 3. Specifically, perceived anthropomorphism of AI was significantly and positively associated with liking of AI (*β* = 0.57, *t* = 15.14, *p* < 0.001), indicating that participants who perceived the AI tool or conversational AI assistant they used most frequently as more human-like tended to report higher levels of liking toward AI. Perceived anthropomorphism of AI was also significantly and positively associated with perceived emotional support (*β* = 0.19, *t* = 4.90, *p* < 0.001). In addition, liking of AI was significantly and positively associated with perceived emotional support (*β* = 0.60, *t* = 15.53, *p* < 0.001), suggesting that users who reported greater liking of AI also tended to perceive stronger emotional support from AI. The model predicting perceived emotional support explained 53% of the variance, R^2^ = 0.53, *F* = 261.88, *p* < 0.001.

**Table 3:** Sequential mediation model of liking of AI and perceived emotional support.

Predictor/Statistic	Liking of AI		Perceived emotional support		Belongingness Need(Total-Effect Model)		Belongingness Need(Full Model)	
	*β*	*t*	*β*	*t*	*β*	*t*	*β*	*t*
Perceived anthropomorphism	0.57	15.14^***^	0.19	4.90^***^	0.34	7.93^***^	0.13	2.47^*^
Liking of AI			0.60	15.53^***^			0.14	2.32^*^
Emotional support							0.25	4.21^***^
*R^2^*	0.33		0.53		0.12		0.21	
*F*	229.15^***^		261.88^***^		62.86^***^		40.57^***^	

*N* = 473. *p < 0.05, **p < 0.01, ***p < 0.00.

When belongingness need was entered as the outcome variable, perceived anthropomorphism of AI was significantly and positively associated with belongingness need before the mediators were included in the model (*β* = 0.34, *t* = 7.93, *p* < 0.001). After liking of AI and perceived emotional support were included in the regression equation, perceived anthropomorphism of AI remained significantly and positively associated with belongingness need (*β* = 0.13, *t* = 2.47, *p* < 0.05). Meanwhile, both liking of AI and perceived emotional support were significantly and positively associated with belongingness need. Specifically, liking of AI was positively associated with belongingness need (*β* = 0.14, *t* = 2.32, *p* < 0.05), and perceived emotional support was also positively associated with belongingness need (*β* = 0.25, *t* = 4.21, *p* < 0.001). The full model explained 21% of the variance in belongingness need, R^2^ = 0.21, *F* = 40.57, *p* < 0.001.

Furthermore, the bootstrap method with 5,000 resamples was used to test the indirect effects, and the results are shown in Table 4. The 95% bootstrap confidence intervals for all three indirect paths did not include zero, indicating that each indirect effect reached statistical significance. Specifically, the indirect association between perceived anthropomorphism of AI and belongingness need through liking of AI was significant, indirect effect = 0.05, BootSE = 0.03, 95% CI [0.01, 0.15], providing support for Hypothesis 2. The indirect association through perceived emotional support was also significant, indirect effect = 0.03, BootSE = 0.02, 95% CI [0.02, 0.08], providing support for Hypothesis 3. In addition, the sequential indirect association from perceived anthropomorphism of AI to belongingness need through liking of AI and then perceived emotional support was significant, indirect effect = 0.05, BootSE = 0.02, 95% CI [0.04, 0.13], providing support for Hypothesis 4.

**Table 4:** Indirect effect analysis.

Indirect path	Effect	Boot SE	Boot LLCI	Boot ULCI
Perceived Anthropomorphism ⇒ Liking of AI ⇒ Belongingness Need	0.05	0.03	0.01	0.15
Perceived Anthropomorphism ⇒ Perceived emotional support ⇒ Belongingness Need	0.03	0.02	0.02	0.08
Perceived Anthropomorphism ⇒ Liking of AI ⇒ Perceived emotional support ⇒ Belongingness Need	0.05	0.02	0.04	0.13

*N* = 473. Bootstrap samples = 5,000. BootSE = bootstrap standard error; BootLLCI = lower limit of the 95% bootstrap confidence interval; BootULCI = upper limit of the 95% bootstrap confidence interval. Indirect effects were considered significant when the 95% bootstrap confidence interval did not include zero.

**Figure 1:**
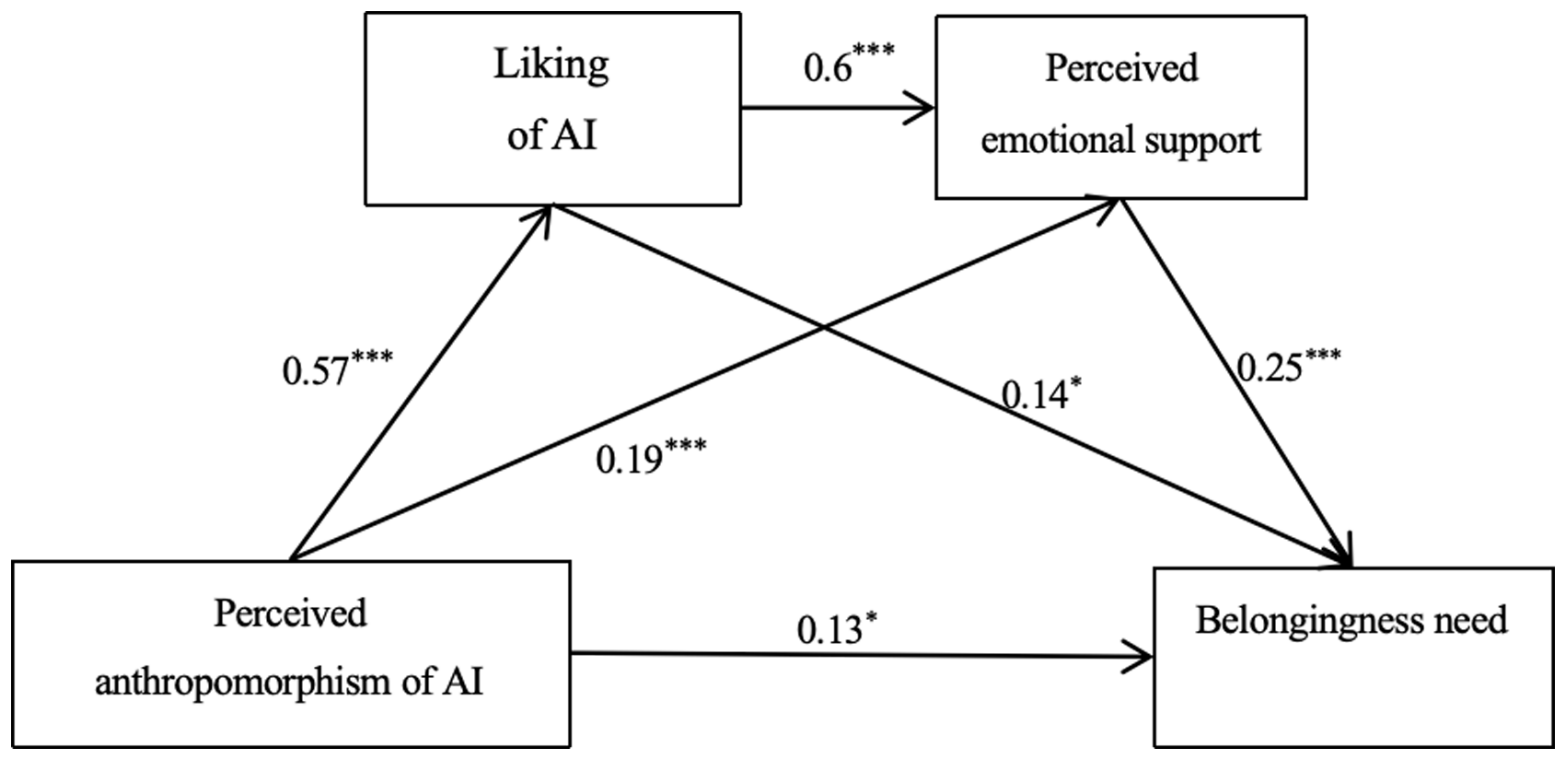
Sequential mediation model.

In conclusion, perceived anthropomorphism of AI was directly associated with belongingness need and was also indirectly associated with belongingness need through multiple indirect paths involving liking of AI and perceived emotional support. More specifically, the results suggest that users who perceived their frequently used AI tool or conversational AI assistant as more human-like tended to report greater liking of AI; this liking was further associated with stronger perceived emotional support, which in turn was associated with higher belongingness need. The overall structure and significant paths of the sequential mediation model are presented in Figure 1. Given the cross-sectional nature of the data, these findings should be interpreted as evidence for a theoretically specified pattern of direct and indirect associations rather than as evidence of causal or temporal effects.

## 4 Discussion

The results of this study revealed a meaningful pattern of associations among perceived anthropomorphism of AI, liking of AI, perceived emotional support, and belongingness need. Specifically, individuals who attributed stronger human-like qualities to the AI tool or conversational AI assistant they used most frequently tended to report stronger belongingness need. At the same time, this association was transmitted through two psychological factors—liking of AI and perceived emotional support—in a sequential mediation model. That is, perceived anthropomorphism of AI was associated with greater liking of AI, which was further associated with stronger perceived emotional support, and perceived emotional support was in turn associated with belongingness need.

This finding is broadly consistent with the three-factor theory of anthropomorphism, which emphasizes the role of social motivation in shaping people’s tendency to attribute human-like qualities to non-human agents (Epley et al., 2007). When social connection is psychologically salient, individuals may become more attentive to social cues in non-human agents and may interpret such agents through a human-like lens. Similarly, the social surrogate hypothesis suggests that people may sometimes turn to non-human or mediated social objects, such as media figures or virtual agents, as psychologically meaningful sources of social experience when social motivation is activated (Gardner and Knowles, 2008; Derrick et al., 2009). In addition, the media equation theory and the CASA paradigm provide a mechanism for understanding why users may respond socially to technologies with anthropomorphic cues: people often apply social rules and interpersonal expectations to media and computer agents, even when they know that these agents are not human (Reeves and Nass, 1996; Nass and Moon, 2000).

The present study extends these perspectives to the context of frequently used AI tools and conversational AI assistants. The findings suggest that anthropomorphic perceptions of AI are not only related to users’ cognitive evaluation of AI as human-like, but also to affective and socioemotional responses, including liking and perceived emotional support. Importantly, however, these results should not be interpreted as evidence that AI objectively satisfies users’ belongingness needs or replaces human interpersonal relationships. The outcome variable in this study assessed the strength of belongingness need, not the degree of belongingness satisfaction. Therefore, the present findings are better understood as showing that anthropomorphic AI perceptions are associated with users’ belongingness-related motivation and socioemotional interpretations of AI interaction. In this sense, the study contributes to human–AI interaction research by clarifying a psychological process linking perceived anthropomorphism, affective appraisal, perceived support, and belongingness need, while also recognizing the conceptual boundary between belongingness need and belongingness fulfillment.

### 4.1 The relationship between perceived anthropomorphism and belongingness need

This study found a significant positive association between perceived anthropomorphism of AI and belongingness need, providing support for Hypothesis 1 at the correlational level. In other words, when users perceived their frequently used AI tool or conversational AI assistant as more human-like, they also tended to report a stronger need for acceptance, connection, and belonging. This result is consistent with the need to belong theory, which proposes that individuals possess a fundamental motivation to form and maintain positive and stable social bonds (Baumeister and Leary, 1995).

This finding can also be understood through the lens of the three-factor theory of anthropomorphism. According to Epley et al. (2007), anthropomorphism is shaped not only by knowledge about human characteristics but also by effectance motivation and sociality motivation. Among these factors, sociality motivation is particularly relevant to the present study. When individuals have stronger belongingness-related motivation, they may be more likely to notice and respond to social cues in AI systems. As a result, AI may be perceived not merely as an instrumental tool, but as a socially meaningful interaction object. This does not mean that AI becomes equivalent to a real interpersonal partner; rather, it suggests that users may interpret AI through social-cognitive processes similar to those used in human interaction.

The CASA paradigm and media equation theory further explain why perceived anthropomorphism may be associated with belongingness need. These theories suggest that users often respond to computers and media technologies in social ways when such systems display human-like or socially responsive cues (Reeves and Nass, 1996; Nass and Moon, 2000). In the context of conversational AI, human-like language style, responsiveness, and perceived understanding may activate users’ social expectations. Consequently, users who perceive AI as more human-like may experience the interaction as more socially relevant, which is associated with stronger belongingness-related psychological tendencies.

At the same time, the present result should be interpreted with caution. Because belongingness need refers to the desire for acceptance and connection, a higher score does not necessarily indicate that users’ belongingness needs have been fulfilled. Thus, the positive association between perceived anthropomorphism and belongingness need may reflect several possible processes. Users with stronger belongingness need may be more likely to anthropomorphize AI; alternatively, perceiving AI as human-like may make users more aware of the social meaning of AI interaction. The cross-sectional design of the present study cannot determine the direction of this association. Therefore, this finding should be understood as preliminary evidence of a meaningful link between anthropomorphic AI perception and belongingness-related motivation, rather than as evidence that AI directly satisfies belongingness needs.

From the perspective of the social surrogate hypothesis, this result suggests that AI may become psychologically relevant in contexts where users’ social motivations are salient. Prior research has shown that media figures, pets, and other non-human agents can sometimes serve as symbolic or temporary social resources (Gardner and Knowles, 2008; Derrick et al., 2009). The present study extends this logic to AI tools and conversational AI assistants, showing that users’ perceptions of AI as human-like are associated with their belongingness-related needs. However, this does not imply that AI can fully substitute for human relationships. Rather, anthropomorphic AI may offer a perceived socioemotional interaction context that is psychologically connected to users’ need for belonging. Future studies should use longitudinal, experimental, or behavioral designs to further clarify whether anthropomorphic AI perceptions precede changes in belongingness-related experiences, or whether users with stronger belongingness needs are more likely to anthropomorphize AI in the first place.

### 4.2 The mediating role of liking

This study found that liking of AI served as a significant mediator in the association between perceived anthropomorphism of AI and belongingness need, providing support for Hypothesis 2. When users perceived their frequently used AI tool or conversational AI assistant as possessing stronger human-like qualities, they tended to report greater liking toward it. This positive affective evaluation was, in turn, associated with stronger belongingness need. This finding suggests that liking may function as an important affective bridge between anthropomorphic perception and belongingness-related motivation.

First, anthropomorphic design tends to increase users’ affection toward AI. A wealth of research shows that products or agents with anthropomorphic features are more likely to receive favorable evaluations and preferences from users (Aggarwal and McGill, 2007). Studies in human–computer interaction have also found that robots with human-like appearances or emotional expressions are more likely to be liked and trusted by users (Eyssel and Hegel, 2012). The reason behind this phenomenon is that anthropomorphism gives otherwise impersonal technologies a stronger sense of social presence. Users may transfer affective habits from human interaction onto anthropomorphized AI and therefore develop more positive feelings toward it.

According to media equation theory and the Computers as Social Actors (CASA) paradigm, when AI displays social cues, users may respond to it in ways that resemble their responses to human interaction partners, including attraction, politeness, trust, and perceived closeness (Reeves and Nass, 1996; Nass and Moon, 2000). For example, in voice-based interactions, AI assistants that adopt a gentle tone or use self-disclosure strategies may encourage users to open up and respond with greater trust and emotional reciprocity (Sun et al., 2025). Therefore, once AI is perceived as more human-like, users may be more likely to assign it a social role and develop positive affective evaluations toward it.

This mechanism can also be explained from the perspective of mental attribution. Anthropomorphism may lead users to attribute thoughts, intentions, or emotional capacities to AI, making it easier for them to perceive AI as an interaction partner rather than as a purely technical system (Waytz et al., 2010). Such perceived mental capacity may increase users’ liking because the AI appears more responsive, approachable, and socially engaging. In this sense, liking is not simply a general preference for technology, but an affective appraisal of AI as a socially meaningful interaction object.

Importantly, liking also helps explain why perceived anthropomorphism may be linked to later perceptions of emotional support. The sequential order proposed in this study is theoretically grounded in the distinction between initial affective appraisal and more relational interpretation. Liking represents a relatively immediate positive evaluation of the AI as pleasant, friendly, and approachable. Perceived emotional support, by contrast, requires users to interpret the AI’s responses as caring, understanding, or comforting. Thus, users may first develop liking toward an anthropomorphized AI; this liking may then make them more willing to interact with the AI, pay attention to its responses, and interpret those responses as emotionally supportive. This provides theoretical justification for placing liking before perceived emotional support in the sequential mediation model.

Liking may also be related to belongingness need because positive affective evaluations often shape how individuals approach potential social objects. In human relationships, liking is one early basis for social approach, interaction willingness, and perceived closeness. Similarly, in human–AI interaction, users who like an AI system may be more willing to engage with it frequently, disclose thoughts to it, and experience the interaction as socially meaningful. However, this does not mean that liking AI directly satisfies belongingness needs or creates the same form of belonging as human relationships. Rather, liking may make AI interaction more psychologically relevant to users’ belongingness-related motivation.

Research on parasocial relationships provides a useful comparison. People often develop one-sided emotional attachments to media characters they like, and such attachments can become psychologically meaningful in moments of loneliness or social disconnection (Derrick et al., 2009). Similarly, when users develop liking toward an AI assistant or social robot, they may begin to experience the AI as part of their broader socioemotional environment, even though the AI is not a real human partner. This perceived socioemotional relevance may help explain why liking of AI was associated with belongingness need in the present study. Nevertheless, because the present data are cross-sectional, this finding should be interpreted as evidence of an indirect association rather than as evidence that liking causally increases belongingness need or fulfills belongingness needs.

### 4.3 The mediating role of emotional support: mechanism explanation

This study also found that perceived emotional support played a significant mediating role in the association between perceived anthropomorphism of AI and belongingness need, providing support for Hypothesis 3. Specifically, users who perceived their frequently used AI tool or conversational AI assistant as more human-like tended to report stronger perceived emotional support from AI, and this perceived support was further associated with stronger belongingness need. This result suggests that emotional support is an important socioemotional pathway through which anthropomorphic perceptions of AI are linked to users’ belongingness-related motivation.

This mechanism aligns closely with classic social support theory. Emotional support generally refers to the perception that an interaction partner provides understanding, empathy, comfort, encouragement, and care—non-material resources that help individuals manage distress and maintain psychological security (Cutrona and Russell, 1990; Cohen, 2004). In interpersonal contexts, feeling heard and comforted by others can make individuals experience a sense of being accompanied and emotionally recognized. In the present study, perceived emotional support from AI should be understood in this subjective sense: it reflects users’ perception that AI responses are supportive, understanding, or comforting, rather than implying that AI possesses genuine human emotions or authentic interpersonal concern.

In the context of human–AI interaction, anthropomorphic perceptions may make such support-related interpretations more likely. When users attribute human-like qualities, awareness, or responsiveness to AI, they may be more inclined to interpret AI replies as considerate, empathic, or emotionally meaningful. This process is primarily enabled by anthropomorphic cues, which give AI perceived social presence and allow users to assign it a quasi-interpersonal role during interaction (Reeves and Nass, 1996; Nass and Moon, 2000). Thus, anthropomorphic AI may not merely be evaluated as a useful tool; it may also be experienced as a source of perceived socioemotional responsiveness.

Prior research on conversational agents, companion chatbots, and health-related technologies provides relevant support for this interpretation (Ta et al., 2020). Studies have shown that virtual agents or chatbots with empathic and emotionally responsive features can be perceived as more caring and supportive, especially when they provide immediate, nonjudgmental responses to users’ concerns (Brave et al., 2005; Hoermann et al., 2017). Similarly, research on assistive robots and healthcare technologies suggests that socially responsive systems may improve users’ perceived acceptance, comfort, or psychological satisfaction in specific contexts (Broadbent et al., 2018). These findings are consistent with the present result that perceived emotional support from AI was positively associated with belongingness need.

At the same time, the present finding should not be interpreted as evidence that AI provides the same form of emotional support as human relationships. Human emotional support is embedded in mutual recognition, shared history, moral responsibility, and reciprocal care, whereas AI-based support is generated through technological interaction and perceived responsiveness. Therefore, the psychological meaning of perceived emotional support from AI may be real for users, but it is not identical to interpersonal support. This distinction is important because the present study measured perceived support and belongingness need, not actual belongingness satisfaction, loneliness reduction, or long-term relational outcomes.

Within the sequential mediation model, perceived emotional support occupies a deeper socioemotional position than liking of AI. Liking reflects an initial positive affective appraisal of AI as pleasant, friendly, or approachable, whereas perceived emotional support reflects users’ interpretation of AI responses as understanding, encouraging, or comforting. Therefore, emotional support represents a more relational layer of human–AI interaction. The significant sequential indirect path found in this study suggests that anthropomorphic perceptions may first be associated with liking, and this positive affective orientation may then make users more receptive to interpreting AI responses as emotionally supportive. In turn, perceived emotional support is associated with stronger belongingness need.

Recent discussions of socioaffective alignment in human–AI relationships further highlight the importance of this mechanism (Kirk et al., 2025). If AI systems are designed to respond in ways that users perceive as emotionally attuned, users may experience the interaction as more socially meaningful. However, such socioaffective alignment should be understood as perceived alignment rather than genuine mutual emotional understanding. The present study therefore contributes to human–AI interaction research by showing that perceived emotional support is not merely a by-product of liking, but a distinct socioemotional mechanism linking anthropomorphic AI perception to belongingness-related motivation. Future research should further examine whether this perceived support leads to changes in actual belongingness satisfaction, loneliness, or relational behavior over time.

### 4.4 Theoretical and practical implications

This study has several theoretical implications. First, it extends anthropomorphism research by applying the three-factor theory of anthropomorphism to the context of frequently used AI tools and conversational AI assistants. Previous research has shown that anthropomorphic cues can shape users’ trust, satisfaction, and acceptance of technological agents. The present study adds to this literature by showing that perceived anthropomorphism is also associated with users’ belongingness-related motivation through affective and socioemotional processes. In this sense, anthropomorphism in AI interaction should not be understood only as a design feature that improves usability or acceptance, but also as a social-cognitive perception that is connected to users’ broader psychological needs.

Second, this study clarifies the psychological process linking perceived anthropomorphism to belongingness need by distinguishing between liking of AI and perceived emotional support. These two constructs are related but not identical. Liking of AI reflects an initial positive affective appraisal of the AI as pleasant, friendly, and approachable, whereas perceived emotional support reflects users’ interpretation of AI responses as understanding, encouraging, and comforting. The significant sequential mediation pathway suggests that anthropomorphic perceptions may first be associated with positive affective evaluation, which is then associated with perceived emotional support. This distinction contributes to human–AI interaction research by showing that users’ socioemotional responses to AI are not a single undifferentiated positive attitude, but may involve layered psychological processes.

Third, the findings refine the application of the social surrogate hypothesis to AI interaction. Prior work on social surrogacy has suggested that media figures, fictional characters, pets, and other non-human agents may become psychologically meaningful when individuals seek connection or social comfort. The present study extends this idea to AI tools and conversational AI assistants, showing that users’ perceptions of AI as human-like are associated with liking, perceived emotional support, and belongingness need. However, this extension should be understood carefully. The findings do not demonstrate that AI provides the same type of belonging as human relationships, nor do they show that AI reduces loneliness or satisfies belongingness needs over time. Rather, they suggest that AI may become a perceived socioemotional interaction object that is psychologically relevant to users’ belongingness-related motivation.

Fourth, the study also contributes to the CASA paradigm and media equation theory. These theories propose that people may apply social rules to computers and media technologies when such technologies present social cues. The present findings suggest that this social response may extend beyond surface-level politeness or usability judgments to include affective appraisal and perceived emotional support. At the same time, the findings also highlight the need to distinguish perceived social responsiveness from genuine interpersonal reciprocity. This distinction is important for developing a more balanced theory of human–AI interaction, one that recognizes users’ real psychological experiences without overstating the relational capacities of AI.

The findings also have practical implications for the design and governance of AI systems. First, designers of conversational AI systems may consider how moderate anthropomorphic cues, such as natural language style, responsiveness, warmth, and clear conversational flow, shape users’ affective evaluations and perceived support. The results suggest that users’ liking of AI and perceived emotional support are important psychological channels through which anthropomorphic perceptions are associated with belongingness-related motivation. Therefore, emotionally sensitive and user-centered interaction design may improve the perceived quality of human–AI interaction.

Second, AI systems intended for educational, counseling-adjacent, or everyday support contexts should be designed with careful attention to perceived emotional support. Responses that acknowledge users’ concerns, provide encouragement, and offer nonjudgmental feedback may help users experience the interaction as more supportive. However, such design should not create the illusion that AI has genuine human emotions or moral responsibility. Transparent communication about the nature and limits of AI support is necessary, especially when users seek emotional comfort or disclose personal concerns.

Third, the findings suggest the importance of calibrating anthropomorphic design. Excessive anthropomorphism may lead users to overestimate AI’s understanding, empathy, or relational capacity, potentially creating unrealistic expectations or emotional dependence. Therefore, AI design should balance warmth with transparency, and social responsiveness with clear boundaries. Rather than simply making AI “more human,” designers should focus on making AI interactions supportive, understandable, and ethically bounded.

Finally, the present findings are relevant for universities and other institutions where students increasingly use AI tools in learning and daily life. Because students may use conversational AI not only for information seeking but also for reassurance, feedback, and emotional expression, institutions should promote AI literacy that includes socioemotional awareness. Users should be encouraged to understand both the potential benefits and limitations of AI-based support, and to maintain human interpersonal relationships as the primary source of deep belonging and reciprocal care.

### 4.5 Limitations and future directions

Despite its contributions, this study has several limitations that should be acknowledged. A primary limitation concerns the cross-sectional nature of the research design. Although the sequential mediation model was developed on the basis of theory, the present data do not allow strong conclusions about temporal order or causal direction. For example, users with stronger belongingness need may be more inclined to anthropomorphize AI, and users who perceive greater emotional support from AI may subsequently develop stronger liking toward it. Therefore, the findings should be interpreted as evidence of theoretically grounded direct and indirect associations rather than as causal effects. Future research could adopt longitudinal designs, cross-lagged panel models, or experimental manipulations of anthropomorphic cues to clarify the directionality of these relationships.

Another important limitation relates to the conceptual interpretation of the outcome variable. The present study measured belongingness need rather than belongingness satisfaction. The Need to Belong Scale captures individuals’ desire for acceptance, connection, and social inclusion, but it does not assess whether this need has been fulfilled. Accordingly, the present findings cannot support the conclusion that anthropomorphic AI satisfies users’ belongingness needs or increases their actual sense of belonging. Future studies should incorporate more direct indicators of belongingness satisfaction, social connectedness, relatedness need satisfaction, loneliness reduction, or perceived social inclusion. Doing so would make it possible to examine whether perceived emotional support from AI is associated not only with stronger belongingness-related motivation, but also with actual changes in users’ social and emotional outcomes.

The study is also limited by the relatively broad way in which AI interaction was operationalized. Participants were instructed to respond with reference to the AI tool or conversational AI assistant they used most frequently, which helped anchor their responses to a concrete interaction object. However, the study did not fully differentiate between specific AI systems or interaction contexts. General-purpose large language model chatbots, voice assistants, customer-service chatbots, learning tools, and companion chatbots may differ substantially in anthropomorphic cues, emotional responsiveness, conversational depth, and user expectations. Future research should therefore collect more detailed information about AI type, primary use purpose, frequency of use, emotional-use experience, and prior familiarity with conversational AI. Such information would help determine whether the proposed mechanism is especially pronounced in emotionally oriented AI interactions, or whether it also applies to more instrumental and task-focused forms of AI use.

Measurement-related issues should also be considered. All variables were assessed using self-report questionnaires collected from the same participants. Although the revised analyses included confirmatory factor analysis and model comparisons to strengthen evidence for measurement validity and construct distinctiveness, self-report data may still be influenced by response style, social desirability, or general affective evaluation. Future studies could combine self-report measures with behavioral indicators, interaction logs, ecological momentary assessment, or experimental observations. For instance, researchers could examine whether users who perceive AI as emotionally supportive actually interact with AI more frequently, disclose more personal concerns, or show different patterns of subsequent help-seeking behavior. In addition, the perceived emotional support scale was adapted from an interpersonal support context to the AI context. Although the adapted scale demonstrated high internal consistency in this study, future research should further validate AI-specific measures of emotional support and examine whether such support includes dimensions such as empathic understanding, encouragement, nonjudgmental response, perceived availability, and personalized feedback.

The generalizability of the findings also requires further examination. The sample consisted of college students from a single cultural context. This population is meaningful for studying human–AI interaction because college students are frequent users of AI tools in learning and daily life. Nevertheless, the extent to which the present findings apply to other age groups, cultural backgrounds, occupational groups, or socially vulnerable populations remains unclear. Future research could examine whether similar mechanisms emerge among adolescents, older adults, working adults, or individuals experiencing loneliness or social exclusion. Cross-cultural research would also help clarify whether users from different sociocultural backgrounds vary in their willingness to anthropomorphize AI or interpret AI responses as emotionally supportive.

Finally, future research should pay closer attention to the boundary conditions and ethical implications of anthropomorphic AI. The present findings suggest that anthropomorphic perceptions may be associated with more positive affective evaluations and stronger perceived emotional support, but these potential benefits should be weighed against possible risks. Excessive anthropomorphism may lead users to overestimate AI’s understanding, empathy, or relational capacity, thereby creating unrealistic expectations, emotional dependence, privacy concerns, or reduced motivation to seek human support when needed. A balanced research agenda should therefore examine not only the socioemotional value of anthropomorphic AI, but also the conditions under which such interactions may become misleading or problematic. From a design perspective, future AI systems should balance warmth with transparency, emotional responsiveness with clear boundaries, and user engagement with protection of autonomy and well-being.

## 5 Conclusion

Grounded in the social and emotional dimensions of human–AI interaction, this study examined how perceived anthropomorphism of AI is associated with users’ belongingness need through liking of AI and perceived emotional support. The findings showed that users who perceived their frequently used AI tool or conversational AI assistant as more human-like tended to report stronger liking of AI and stronger perceived emotional support, both of which were associated with belongingness need. The significant sequential mediation pathway further suggests that anthropomorphic perceptions may be linked to belongingness-related motivation through a layered psychological process, moving from affective appraisal to perceived socioemotional support.

This study contributes to human–AI interaction research by clarifying that users’ responses to anthropomorphic AI are not limited to instrumental evaluation or functional acceptance. Rather, anthropomorphic perceptions may also be associated with affective and socioemotional interpretations of AI interaction. In particular, the distinction between liking of AI and perceived emotional support helps explain how users’ positive evaluations of AI may develop into a more relational perception of AI as supportive or understanding. At the same time, the present findings should be interpreted within their conceptual and methodological boundaries. The outcome variable measured belongingness need rather than belongingness satisfaction; therefore, the results do not demonstrate that AI fulfills users’ belongingness needs or replaces human relationships.

Overall, the study suggests that anthropomorphic AI may become psychologically meaningful in users’ socioemotional experience, especially when users perceive AI as friendly, responsive, and emotionally supportive. For designers and practitioners, the findings highlight the importance of developing AI systems that are warm, understandable, and responsive while maintaining transparency about their artificial nature and limitations. Future research should further examine whether and under what conditions perceived emotional support from AI contributes to actual belongingness satisfaction, social connectedness, loneliness reduction, or long-term human–AI relational outcomes.

## Data Availability

The raw data supporting the conclusions of this article will be made available by the authors, without undue reservation.
